# A Lipidated Single-B-Chain Derivative of Relaxin Exhibits Improved In Vitro Serum Stability without Altering Activity

**DOI:** 10.3390/ijms24076616

**Published:** 2023-04-01

**Authors:** Praveen Praveen, Chao Wang, Thomas N. G. Handley, Hongkang Wu, Chrishan S. Samuel, Ross A. D. Bathgate, Mohammed Akhter Hossain

**Affiliations:** 1 Florey Institute of Neuroscience and Mental Health, University of Melbourne, Parkville, VIC 3052, Australia; pinki0807@gmail.com (P.P.); hongkangw@student.unimelb.edu.au (H.W.); 2 Cardiovascular Disease Program, Monash Biomedicine Discovery Institute, Department of Pharmacology, Monash University, Clayton, VIC 3168, Australia; chao.wamg@monash.edu (C.W.); chrishan.samuel@monash.edu (C.S.S.); 3 Department of Biochemistry and Pharmacology, Florey Institute of Neuroscience and Mental Health, University of Melbourne, Parkville, VIC 3010, Australia; bathgate@florey.edu.au; 4 Florey Department of Neuroscience and Mental, Florey Institute of Neuroscience and Mental Health, School of Chemistry, Department of Biochemistry and Pharmacology, University of Melbourne, Parkville, VIC 3010, Australia

**Keywords:** H2 relaxin, RXFP1, B7-33, structure-activity relationship (SAR)

## Abstract

Human relaxin-2 (H2 relaxin) is therapeutically very important due to its strong anti-fibrotic, vasodilatory, and cardioprotective effects. Therefore, relaxin’s receptor, relaxin family peptide receptor 1 (RXFP1), is a potential target for the treatment of fibrosis and related disorders, including heart failure. H2 relaxin has a complex two-chain structure (A and B) and three disulfide bridges. Our laboratory has recently developed B7-33 peptide, a single-chain agonist based on the B-chain of H2 relaxin. However, the peptide B7-33 has a short circulation time in vitro in serum (t_1/2_ = ~6 min). In this study, we report structure-activity relationship studies on B7-33 utilizing different fatty-acid conjugations at different positions. We have shown that by fatty-acid conjugation with an appropriate spacer length, the in vitro half-life of B7-33 can be increased from 6 min to 60 min. In the future, the lead lipidated molecule will be studied in animal models to measure its PK/PD properties, which will lead to their pre-clinical applications.

## 1. Introduction

The human gene-2 relaxin peptide (H2 relaxin) was discovered as a hormone of reproduction [1]. The actions of H2 relaxin during pregnancy are pleiotropic, including strong antifibrotic, angiogenic, vasodilatory, antiapoptotic, and anti-inflammatory effects [2,3,4]. These effects are mediated through the activation of the G protein-coupled receptor, Relaxin Family Peptide Receptor 1 (RXFP1), the native receptor for H2 relaxin [5]. However, H2 relaxin also has a high affinity for, and potency on, the related Relaxin Family Peptide Receptor 2 (RXFP2, a cognate receptor for INSL3 [6]) and the glucocorticoid receptor [7], which may lead to off-target effects. Additionally, due to its complex two (A and B)-chain structure that is connected via three disulfide bridges, H2 relaxin is very time-consuming, tedious, laborious, and expensive to produce, let alone to modify to improve its poor pharmacokinetic properties. Therefore, the design and development of a peptidomimetic of H2 relaxin has been long sought after, one that is easily amenable to modifications and large-scale production. However, the complex mechanism of the H2-relaxin–RXFP1 interaction indicates significant challenges in modifying and minimizing the peptide size without altering its biological activity [8]. After over a decade of structure–activity relationship studies on both the peptide ligand and receptor, our group was finally able to develop a single-chain derivative of H2 relaxin, B7-33 [9] that was shown to be biologically active. B7-33 was tested in rat renal myofibroblasts (from injured kidneys) and human cardiac fibroblasts (endogenously expressing RXFP1 cells) for its ability to promote MMP-2, a collagen-degrading enzyme. B7-33 exhibited a similar MMP-2 production ability to H2 relaxin in these cells [9]. Most importantly, like H2 relaxin, B7-33 reversed or resolved fibrosis in several animal models tested to date (rat vs. mouse; heart fibrosis vs. lung fibrosis) [9,10,11,12]. It is also interesting to observe that B7-33 did not activate all the signalling pathways of H2 relaxin in every cell we tested and that the signalling mechanism seemed to be cell-specific [9,13]. In fibroblasts, while it was shown to barely activate cyclic adenosine monophosphate (cAMP), which is postulated to be associated with tumor growth [9], B7-33 activated the pERK pathway with a potency similar to H2 relaxin [9], and pERK activation is known to link to the anti-fibrotic and vasodilatory effects of H2 relaxin [4,14].

**Figure 1:**
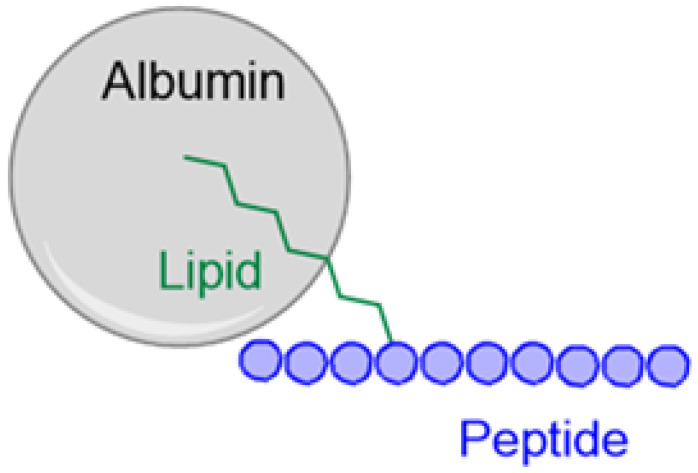
Schematic representation of fatty acid interacting with albumin. Lipid interacts non-covalently to albumin.

A significant limitation of peptides as drug candidates is their short half-life [15,16], as they are rapidly degraded in vivo by enzymes and excreted and metabolized by the kidneys and liver [15,17]. The in vivo half-life of H2 relaxin is approximately ten minutes [18,19]. Therefore, continuous intravenous infusion into patients over 48 h is required for it to demonstrate efficacy [20]. B7-33 is an unstructured single-chain peptide [9], and consequently, its in vivo half-life is expected to be even less than H2 relaxin. Recently Mallart et al. developed potent and long-acting B7-33 analogues [21]. However, these peptide variants, unlike B7-33, activated the cAMP pathway, which might have detrimental effects including tumor growth. Therefore, this study focuses on B7-33, which exhibited signalling biased towards pERK over cAMP, and describes modifications to enhance the half-life of B7-33 by using a lipidation strategy that was developed in the mid-1990s [22]. Lipid-conjugated peptides interact with albumin at three high-affinity interaction sites, between domains IIA and IIB, and within domain III [23]. Therefore, because of their bulky size, they get excreted slower (Figure 1). Albumin is the most abundant protein in plasma, it has a half-life of 19 days in humans, and its size is above the renal filtration size [24]. Today, the most well-known lipidated peptide drugs on the market are detemir (Levemir^®^) [25], degludec (Tresiba^®^) [26], liraglutide (Victoza^®^) [27], and semaglutide [28]. We have used the following three strategies to lipidate B7-33:

(1) Different chain lengths of fatty acids,

(2) Solubilizing tags,

(3) Different lengths of spacers between the fatty acid and B7-33.

## 2. Results and Discussion

Peptides were synthesized and conjugated with PEG and fatty-acid moieties by standard fmoc synthesis on solid support, and the details are described in the experimental section.

### 2.1. Different Lengths of Fatty Acids

**Figure 2:**
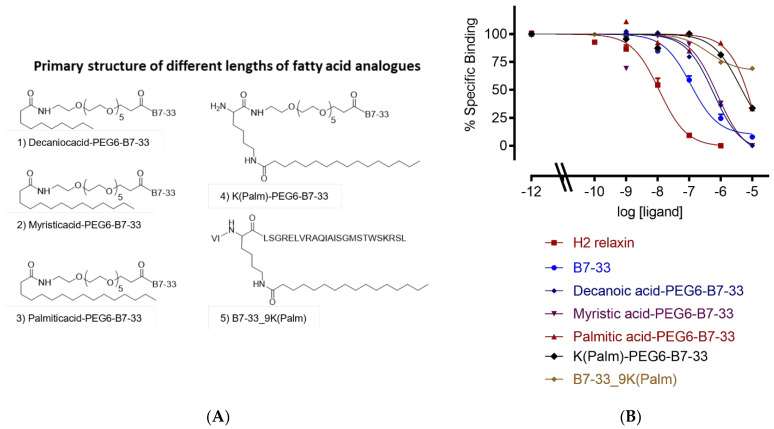
Primary structure and sequence of lipidated analogues. (**A**) Different lengths of fatty acids: (1) decanoic acid (C10), (2) myristic acid (C14), (3) palmitic acid (C16), (4) lysine palmitic acid (C16), and (5) lysine replaced with lysine palmitic acid at 9th position of B7-33. (**B**) Eu-H2 relaxin competition binding of lipidated analogues in HEK-7BP cells. The data are the result of *n* = 3–4 independent experiments and expressed as mean ± SEM.

We conjugated decanoic acid (C10), myristic acid (C14), and palmitic acid (C16) at the N-terminus of B7-33 with PEG6 as a spacer, so that the fatty acid would not interfere with the binding sites of B7-33 (Figure 2). We also synthesized one analogue where palmitic acid was conjugated at the side chain of lysine 9 of B7-33 (Figure 2A, 5). We carried out competition binding assays in HEK-293T cells stably expressing the 7BP protein, a surrogate for RXFP1 (details are described in the experimental section). Binding data suggest (Figure 2B, Table 1) that increasing the carbon length of fatty acids decreases their binding affinity to the RXFP1 ectodomain. The data suggest that the ten-carbon decanoic acid (C10) derivative of B7-33 has a stronger binding affinity than fourteen-carbon myristic acid (C14) and sixteen-carbon palmitic acid (C16). This is possibly due to the hydrophobic nature of palmitic acid, or we need a longer spacer than PEG6 that can separate the fatty acids from B7-33.

### 2.2. Solubilizing Tag

**Figure 3:**
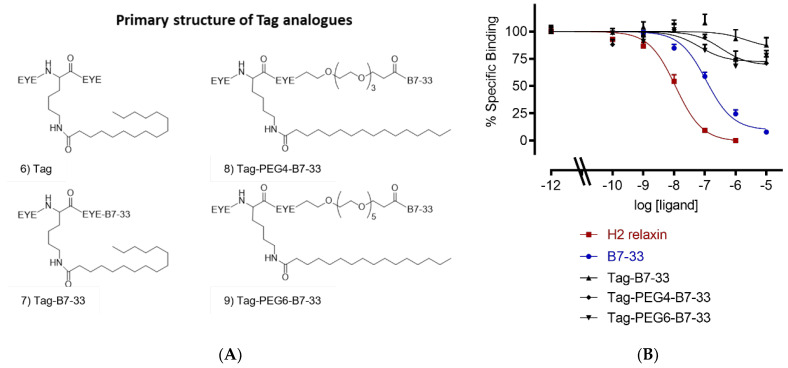
Primary structure and sequence of lipidated analogues. (**A**) Primary structure of tagged B7-33 analogues: (6) Tag structure, (7) Tag-B7-33 with no spacer, (8) Tag-PEG4-B7-33 with PEG4 spacer, and (9) Tag-PEG6-B7-33 with PEG6 spacer. (**B**) Eu-H2 relaxin competition binding of apidated analogues in HEK-7BP cells. The data are the result of *n* = 3–4 independent experiments and expressed as mean ± SEM.

As fatty acid analogues were challenging to solubilize, we used a tag heptapeptide (Figure 3A) developed by Zorzi et al. [29]. It was shown that this tag peptide improved the proteolytic stability of a series of peptides [29]. It prolonged the circulation time of the peptides in rats and rabbits around 24-fold, increasing their half-lives for 5 to 7 h [29]. We made three tagged B7-33 analogues in this study (Figure 3A, 7–9). However, all tagged B7-33 analogues showed a reduced binding affinity (Figure 3B, Table 1). Therefore, we decided to investigate the length of the PEG spacer.

### 2.3. Different Lengths of Spacer

**Figure 4:**
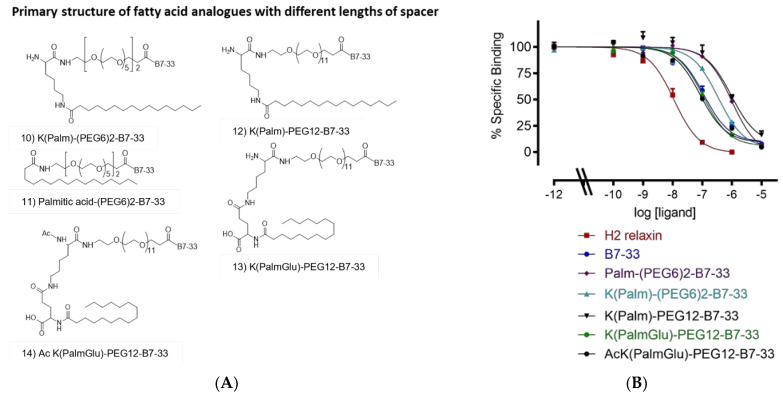
Primary structure and sequence of lipidated analogues. (**A**) Primary structure and sequence of lipidated analogues with different lengths of spacer used between the fatty acid and B7-33. (**B**) Eu-H2 relaxin competition binding of lipidated analogues in HEK-7BP cells. The data are the result of *n* = 3–4 independent experiments and expressed as mean ± SEM.

We employed a longer PEG spacer between the fatty acid and B7-33 (Figure 4). We used two PEG6 spacers in K(Palm)-(PEG6)2-B7-33 and Palmitic acid-(PEG6)2-B7-33 (Figure 4A, 10 and 11), PEG12 in K(Palm)-PEG12-B7-33 (Figure 4A, 12), and PEG12 and glutamic acid as a spacer in K(PalmGlu)-PEG12-B7-33 (Figure 4A, 13). We acetylated the K(PalmGlu)-PEG12-B7-33 analogue to provide resistance to proteolytic degradation (Figure 4A, 14). Binding studies showed that AcK(PalmGlu)-PEG12-B7-33 is the best-lipidated analogue with similar binding as B7-33 to the RXFP1 ectodomain (Figure 4B, Table 1). We used this peptide, AcK(PalmGlu)-PEG12-B7-33, as a lead to study its stability in vitro.

### 2.4. In Vitro Serum Stability of AcK(PalmGlu)-PEG12-B7-33

**Figure 5:**
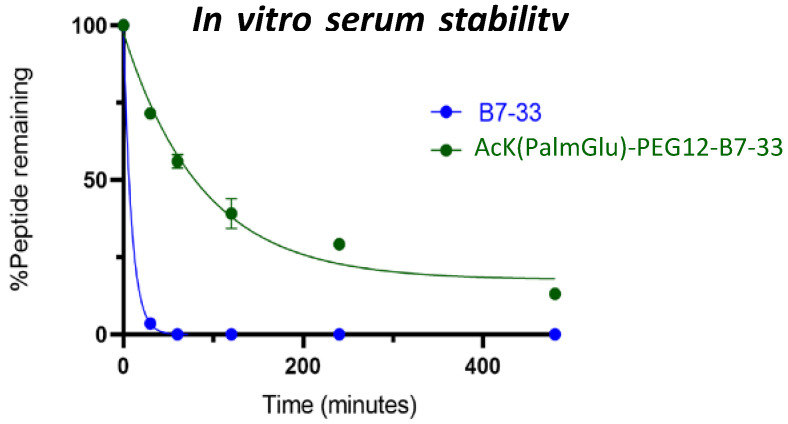
In vitro serum stability assay. B7-33 degrades within minutes, whereas AcK(PalmGlu)-PEG12-B7-33 is stable in serum over time. There were significant differences between the peptides at any time point (*p*-value = 0.0222, *t*-test). One-phase exponential decay and paired t-test analyses were performed with GraphPad Prism 8.4.3. The data are the results of *n* = 3 independent experiments.

In vitro serum stability studies (*n* = 3) showed that B7-33 linear peptide degrades quickly (within minutes), whereas AcK(PalmGlu)-PEG12-B7-33 peptide exists in serum in vitro for a longer time (Figure 5). The in vitro half-life of B7-33 and AcK(PalmGlu)-PEG12-B7-33 is 6 and 60 min, respectively (Figure 5). The presence of the fatty acid increased the in vitro half-life of B7-33. This increased stability in vitro suggests that this may reduce the need for large doses and frequent injections of the peptide to maintain its therapeutic effect. This needs to be investigated in in vivo animal models.

### 2.5. The Effects of Lipdated B7-33 (L-B7-33), AcK(PalmGlu)-PEG12-B7-33 on Myofibroblast Differentiation In Vitro

**Figure 6:**
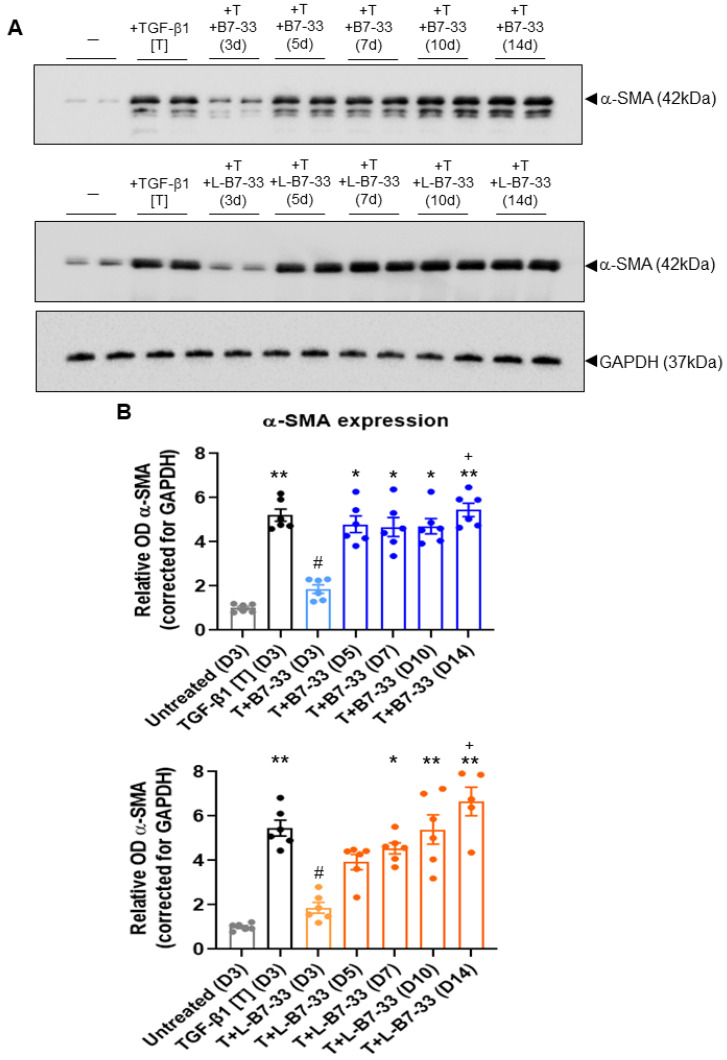
The effects of B7-33 vs. L-B7-33 on myofibroblast differentiation in vitro. (**A**) Representative Western blots of α-SMA expression and corresponding GAPDH expression from unstimulated BJ3 human dermal fibroblasts, TGF-β1 (T)-stimulated BJ3 human dermal myofibroblasts and T + B7-33- or T + L-B7-33-treated myofibroblasts after 3 days (D3); and T + B7-33- or T + L-B7-33-treated myofibroblasts after 5 days (D5), 7 days (D7), 10 days (D10) or 14 days (D14). (**B**) Also shown are the mean ± SEM levels of a-SMA expression from each treatment group, corrected for GAPDH loading and expressed relative to the unstimulated cell control group, which was expressed as 1 in each case; from *n* = 6 separate experiments conducted in duplicate. Statistical analysis: *p* < 0.05, * *p* < 0.01 vs. untreated (D3) group; ^#^ *p* < 0.05 vs. TGF- b1 [T] (D3) group; ^+^ *p* < 0.05 vs. T+B7-33 or T+L-B7-33 (D3) group, as determined using a non-parametric Kruskal Wallis test and Dunn’s post-hoc analysis to allow for multiple comparisons between the groups.

To determine if the lipidation of B7-33 prolonged its ability to suppress myofibroblast differentiation in vitro, the effects of the native B7-33 peptide vs. AcK(PalmGlu)-PEG12-B7-33 (we call it “L-B7-33”) were compared in TGF-α (T)-stimulated BJ3 human dermal myofibroblasts (Figure 6). T (2 ng/mL) alone stimulated a ~4–4.5-fold increase in α-SMA expression (as a measure of myofibroblast differentiation) after 72 h of culture. This TGF-α-induced increase in α-SMA expression was almost fully suppressed by the co-administration of either native B7-33 (100 ng/mL) or L-B7-33 (100 ng/mL) after 72 h of culture (both *p* < 0.05 vs. T-alone group; both no different to the unstimulated cell group; Figure 6). However, in the presence of native B7-33 treatment, α-SMA expression levels fully returned to those measured for T-alone-stimulated cells, after 5–14 days of culture (all *p* < 0.05 vs. unstimulated group; all no different to T-alone group; Figure 6). Comparatively, in the presence of L-B7-33 treatment, α-SMA expression levels returned more gradually to those measured for T-alone-stimulated cells, so that they returned to ~70% of those measured in T-alone-stimulated cells after 5 days (no different to unstimulated cell group; no different to the T alone group); but then they fully returned to those measured for T-alone-stimulated cells after 7–14 days of culture (all *p* < 0.05 vs. unstimulated cell group; no different to the T alone group; Figure 6). In each case, α-SMA expression levels that were measured after 14 days of B7-33 or L-B7-33 treatment were significantly higher than those measured after 3 days of peptide treatment (*p* < 0.05 vs. B7-33 or L-B7-33 treatment after 3 days, respectively; Figure 6). These findings demonstrate the proof of principle that the effects of L-B7-33 are more prolonged than the effects of native B7-33 in inhibiting myofibroblast differentiation in vitro.

## 3. Materials and Methods

### 3.1. Materials

9-fluoroenylmethoxycarbonyl (Fmoc), O-(1H-6-chlorobenzotriazole-1-yl)-1,1,3,3-tetramethyluronium hexafluorophosphate (HCTU)/1-[Bis(dimethylamino)methylene]-1H-1,2,3-triazolo[4,5-b]pyridinium 3-oxide hexafluorophosphate (HATU) were purchased from GL Biochem (Shanghai, China). Rink Amide-MBHA Resin (substitution 0.285 mmol/g and substitution 0.36 mmol/g) was purchased from GL Biochem (Shanghai, China). Trifluoroacetic acid (TFA) was obtained from Auspep (Melbourne, Australia). Acetonitrile, dichloromethane, piperidine, diethyl ether, N,N′-dimethylformamide (DMF) and methanol were from Merck, Melbourne, Australia. Palmitic acid, myristic acid, decanoic acid, TIPS (triisopropylsilane), anisol, sinapinic acid (3,5-dimethyl-4-hydroxycinnamic acid), DHB (2,5-dihydroxybenzoic acid), TFE (2,2,2-Trifluoroethanol), and human serum were purchased from Sigma-Aldrich, Melbourne, Australia. Fmoc-L-Lys(Palm-L-Glu-OtBu)-OH and Fmoc-L-Lys(Palm)-OH were purchased from Iris Biotech GMBH; Fmoc-NH-PEG-COOH, Fmoc-NH-(PEG)3-COOH, Fmoc-NH-(PEG)5-COOH and Fmoc-NH-(PEG)11-COOH were purchased from Merk-millipore. All other reagents were from Sigma-Aldrich, Melbourne, Australia.

### 3.2. Solid-Phase Peptide Synthesis

All the single-chain peptides were synthesized using Fmoc solid-phase synthesis on a microwave-assisted liberty peptide synthesizer (CEM Liberty, Mathew, USA) or manually using Fmoc-protected L-α-amino acids on Rink amide resins. All side chain-protecting groups of amino acids were TFA-labile. The peptides were synthesized on either a 0.1 or 0.2 mmol scale using instrument default protocols or manually with a 4-fold molar excess of Fmoc-protected amino acids activated by a 4-fold excess of HCTU/HATU in the presence of an excess (6-fold) of diisopropylethelene amine (DIEA). Nα-Fmoc protecting groups were removed by treating the resin-attached peptide with piperidine (20% *v*/*v*) in DMF. Using the microwave synthesizer, the coupling and deprotection were carried out at 75 °C using 25 W microwave power for 5 min and 60 W microwave power for 5 or 3 min, respectively. For manual synthesis, the coupling and deprotection were carried out for 60 min and 5 min three times, respectively.

### 3.3. Peptide Cleavage from Solid Support

After completion of the solid phase synthesis, peptides were cleaved by using a cleavage cocktail of trifluoroacetic acid (TFA): TIPS (triisopropylsilane): water: Anisol (94:1:2:3) for 2 h. The cleavage solution was filtered using a filter and evaporated under nitrogen. The cleaved peptide was precipitated in cold ether and centrifuged for 5 min; this step was repeated at least four times.

### 3.4. Peptide Purification and Characterization

An analytical RP-HPLC analysis of the purified peptides was performed with Waters RP-HPLC systems. Empower software was used for data collection, monitoring, and analysis. The RP-HPLC profiles were acquired using a Phenomenex Gemini C18 analytical column (4.6 × 250 mm, pore size 110 Å, particle size 5 μm) at a constant flow rate of 1.5 mL/min, in a gradient mode with buffer A, 0.1% TFA in water, and buffer B, 0.1% TFA in acetonitrile, monitoring at a wavelength of 214 nm, which is characteristic for the amide bond. All HPLC purifications were performed using a Phenomenex C18 preparative column (21.2 × 150 mm) at a constant flow rate of 10 mL/min, in a gradient mode with eluent A, 0.1% TFA in water, and eluent B, 0.1% TFA in acetonitrile.

Peptide characterization was carried out on a Bruker Ultraflex II instrument (Bruker Daltonics, Bremen, Germany) MALDI-TOF MS (matrix-assisted laser desorption ionization time-of-flight mass spectrometry). Sinapinic acid (3,5-dimethyl-4-hydroxycinnamic acid) and DHB (2,5-dihydroxybenzoic acid) in 0.1% TFA in A.C.N./water (7:3) were used as the matrix. The traces from HPLC and MALDI-TOF MS for purified 14 peptides (Figures S1–S14) are available in the supporting information.

### 3.5. Amino Acid Analysis

The peptide content was determined using Direct Detect^®^ assay-free sample cards and the Direct Detect^®^ spectrometer. Each card contains hydrophilic spots surrounded by a hydrophobic ring to retain the analyzed sample within the IR beam for convenient sample application and analysis. All measurements were performed using 2 μL of the sample solution.

### 3.6. HEK-7BP Binding Assays

HEK-293T cells stably expressing the 7BP protein (HEK-7BP; RXFP1 extracellular domain attached with a single TM domain instead of the native 7 TM domain [30]) were plated in 96-well plates. The medium was aspirated off, and the cells were washed with phosphate-buffered saline (PBS) before competition binding assays were performed with Eu-H2 Relaxin (RXFP1 ligand), as previously described [31], with the only difference being that BSA was excluded from the binding assay buffer. Competition binding curves for each peptide were performed in triplicate, and each experiment was performed independently at least three times. Fluorescent measurements were carried out on a BMG POLARstar plate reader (BMG Labtech, Melbourne, Australia). Pooled data are presented as mean ± SE of specific binding and are fitted using a one-site binding curve in GraphPad Prism version 8 (GraphPad Inc., San Diego, CA, USA). Statistical analyses were conducted using a one-way analysis of variance with uncorrected Fisher’s least significant difference (LSD) post-hoc analysis in GraphPad Prism version 8.

### 3.7. In Vitro Serum Stability Assay

A total of 400 µL of human serum was taken, and 200 µg peptide dissolved in 100 µL of water was added to human serum at 37 °C. A total of 80 µL of samples was removed at various time points (0 min, 30 min, 60 min, 120 min, 240 min, and 480 min), and 160 µL of acetonitrile was added to precipitate plasma proteins. The solution was then centrifuged at 12,000 rpm for 15 min at 4 °C. A total of 200 µL of supernatant was taken out and mixed with 240 µL of milli-Q water, and each sample was analyzed by RP-HPLC (2 × 80 μL injection), by measuring the area under the peak at the appropriate retention time compared to the peak area of the peptide at time zero minute (100%). Data analysis (*n* = 3, duplicate) was performed using Prism Version 8.0.2, a non-linear-fit one-phase decay model.

### 3.8. Determination of the Effects of Lipidated (L)-B7-33 on Myofibroblast Differentiation In Vitro

To determine if the lipidation of B7-33 prolonged its ability to suppress myofibroblast differentiation in vitro, the effects of the native B7-33 peptide vs. lipidated (L)-B7-33 were compared in BJ3 cells, a human dermal fibroblast cell line [1,32]. BJ3 fibroblasts were used between passages 15–18 and cultured as described before [2,33]. Under the experimental conditions detailed below, the fetal calf serum (FCS) content of the media used was reduced from 15% down to 0.5% (as FCS acts as a protein carrier for B7-33 activity, this maintains its activity over the time course during which B7-33 is exposed to cells in vitro; hence, the long-term activity of B7-33 was expected to be minimized in the presence of 0.5% FCS). BJ3 cells were plated in 12-well plates at a density of 1–1.25 × 10^5^ cells per well and were then either left untreated or stimulated with transforming growth factor (TGF)-β1 (T; 2 ng/mL) alone (to promote myofibroblast differentiation) or with T + B7-33 (100 ng/mL) or T + L-B7-33 (100 ng/mL) for a 14-day period, in media that was left unchanged over this time period. The cells from duplicate wells per treatment group were isolated from plates at 3-, 5-, 7-, 10- and 14-days post-T ± peptide treatment and extracted for protein using the RIPA lysis buffer (ThermoFisher Scientific; Scoresby, Victoria, Australia). These experiments were performed six independent times.

Half the cell protein (in 10 μL of RIPA buffer) from each sample (that was fully reconstituted in 20 μL of RIPA buffer) was loaded and electrophoresed in separate mini-protean 4–15% precast gels, and analyzed by Western blotting using a primary monoclonal antibody to α-SMA (42 kDa; M0851; 1:000 dilution; Agilent Technologies, Santa Clara, CA, USA) [34,35]. The equivalent loading of protein between samples was confirmed using a rabbit monoclonal glyceraldehyde 3-phosphate dehydrogenase (GAPDH) housekeeping antibody (37 kDa; 2118; 1:1000 dilution; Cell Signaling Technology; Danvers, MA, USA). Each membrane was then subjected to appropriate anti-mouse (7076) or anti-rabbit (7074) horseradish peroxidase-conjugated secondary antibodies (both at a 1:2000 dilution; Cell Signaling Technologies), before proteins were detected using the Clarity Western ECL substrate detection kit and quantified by densitometry with a ChemiDoc MP Imaging System and Image Lab v.6.0 software (both from Bio-Rad Laboratories, Hercules, CA, USA).The density of α-SMA from each sample was corrected for GAPDH protein levels and then expressed relative to the ratio that was measured from the unstimulated control group, which was expressed as 1 in each case. Representative blots of α-SMA and GAPDH were also chosen for presentation in each case.

## 4. Conclusions

In conclusion, we have shown that by fatty-acid conjugation with an appropriate spacer length, the in vitro half-life of B7-33 can be increased from 6 min to 60 min. These analogues will be studied in animal models to measure their PK/PD properties in the future.

## Figures and Tables

**Table 1:** List of analogues and their binding affinities (pKi) in HEK-7BP cells, BSA free assay.

Analogue	HEK-7BP Binding B.S.A. Free Eu-H2 pKi (*n*)
B7-33	7.28 ± 0.11 (7)
Decanoic acid-PEG6-B7-33	6.63 ± 0.14 (3)
Myristic acid-PEG6-B7-33	6.0 ± 0.11 (3) **
Palmitic acid-PEG6-B7-33	<5 (3)
K(Palm)-PEG6-B7-33	5.71 ± 0.14 (3) ***
B7-33_9K(Palm)	<5 (3)
Tag-B7-33	<5 (3)
Tag-PEG4-B7-33	<5 (3)
Tag-PEG6-B7-33	<5 (3)
K(Palm)-(PEG6)2-B7-33	6.80 ± 0.16 (3)
PA-(PEG6)2-B7-33	6.40 ± 0.10 (3) **
K(Palm)-PEG12-B7-33	6.56 ± 0.52 (3) **
K(PalmGlu)-PEG12-B7-33	7.37 ± 0.10 (3)
Ac K(PalmGlu)-PEG12-B7-33	7.52 ± 0.13 (3)

** *p* < 0.01; *** *p* < 0.001 vs. B7-33.

## Data Availability

All the data are the result of *n* = 3–4 independent experiments and expressed as mean ± SEM. We used GraphPad Prism 9 to make graphs and statistically analyze data.

## Supplementary Materials

The supporting information can be downloaded at: https://www.mdpi.com/article/10.3390/ijms24076616/s1.
