# Supplementary material for: A Lipidated Single-B-Chain Derivative of Relaxin Exhibits Improved In Vitro Serum Stability without Altering Activity
Source: Int J Mol Sci. 2023 Apr 1;24(7):6616. doi: 10.3390/ijms24076616 (PMC10094921; doi:10.3390/ijms24076616)
Supplement: Supplementary file 1 [file ijms-24-06616-s001.zip › ijms-2304952-supplementary.pdf]

SUPPORTING INFORMATION

1.1.1 Analytical RP-HPLC and MALDI-TOF-MS of all compounds

1. B7-33

RP-HPLC carried out as described in the methods using the elution gradient: buffer B 20-50% in 30 minutes, t= 17.081 min.

MALDI-TOF-MS $[M+H]^+$ calculated 2986.555, observed 2990.834.

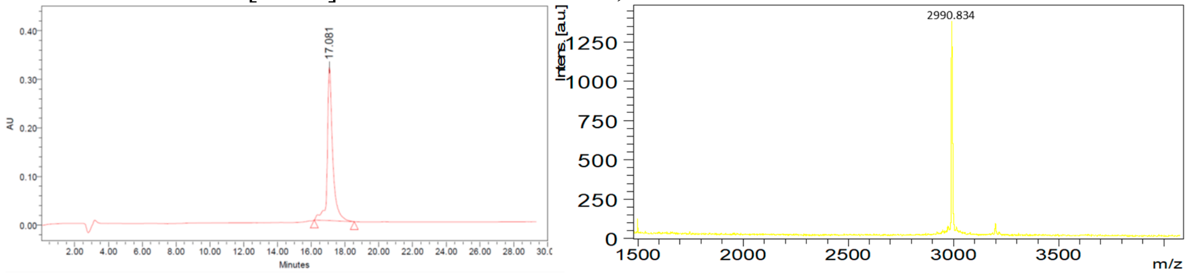

Figure S1: RP-HPLC profile of B7-33 (left) and MALDI-TOF-MS (right).

2. Decanoic acid-PEG6-B7-33

RP-HPLC carried out as described in the methods using the elution gradient: buffer B 35-65% in 30 minutes, t= 12.229 min.

MALDI-TOF-MS $[M+H]^+$ calculated 3475.425, observed 3481.099.

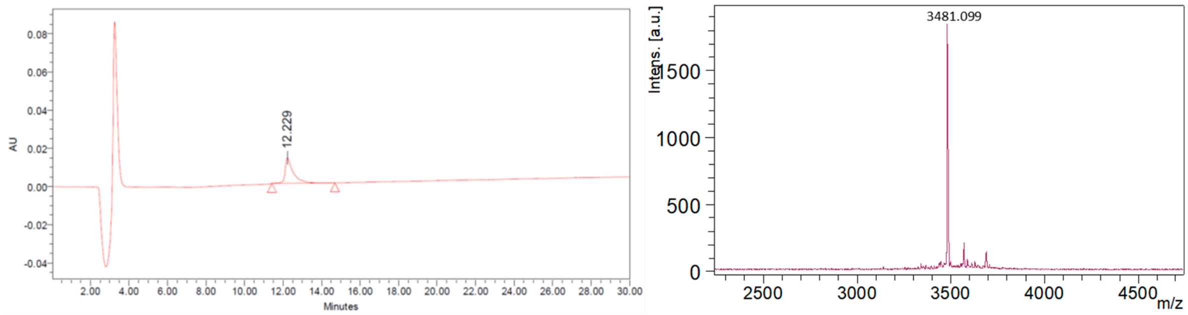

Figure S2: RP-HPLC profile of Decanoic acid-PEG6-B7-33 (left) and MALDI-TOF-MS (right).

3. Myristic acid-PEG6-B7-33

RP-HPLC carried out as described in the methods using the elution gradient: buffer B 35-65% in 30 minutes, t= 17.365 min.

MALDI-TOF-MS $[M+H]^+$ calculated 3532.535, observed 3536.314.

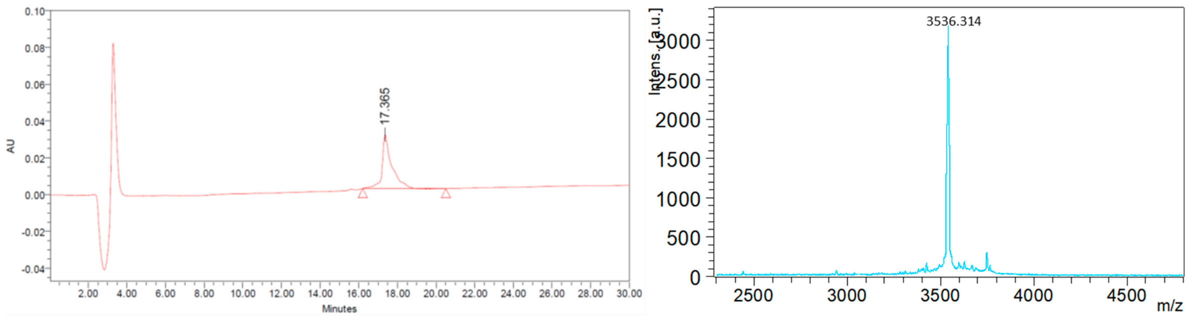

Figure S3: RP-HPLC profile of Myristic acid-PEG6-B7-33 (left) and MALDI-TOF-MS (right).

4. Palmitic acid-PEG6-B7-33

RP-HPLC carried out as described in the methods using the elution gradient: buffer B 35-65% in 30 minutes, t= 20.330 min.

MALDI-TOF-MS $[M+H]^+$ calculated 3560.585, observed 3559.294.

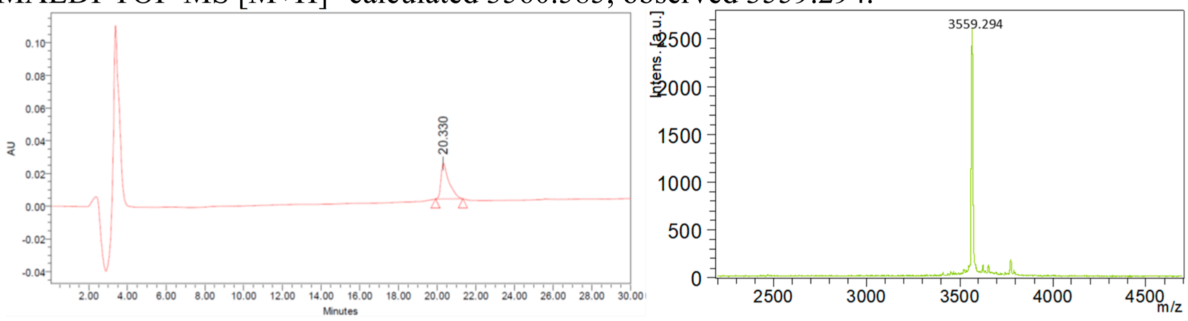

Figure S4: RP-HPLC profile of Palmitic acid-PEG6-B7-33 (left) and MALDI-TOF-MS (right).

5. K(Palm)-PEG6-B7-33

RP-HPLC carried out as described in the methods using the elution gradient: buffer B 30-60% in 30 minutes, t= 23.823 min.

MALDI-TOF-MS $[M+H]^+$ calculated 3689.015, observed 3691.177.

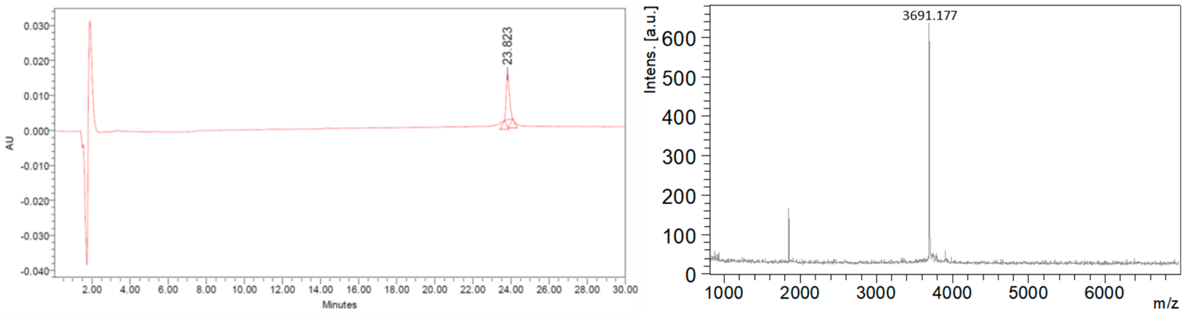

Figure S5: RP-HPLC profile of K(Palm)-PEG6-B7-33 (left) and MALDI-TOF-MS (right).

6. B7-33_9K(Palm)

RP-HPLC carried out as described in the methods using the elution gradient: buffer B 30-60% in 30 minutes, t= 26.347 min.

MALDI-TOF-MS $[M+H]^+$ calculated 3225.231, observed 3229.264.

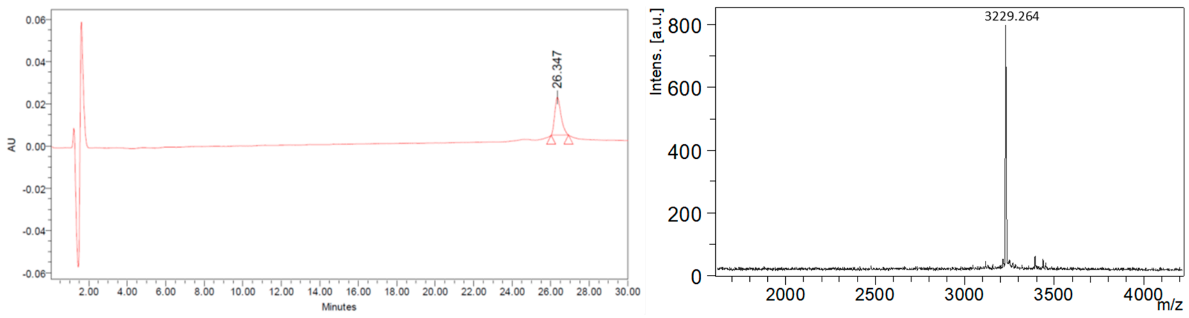

Figure S6: RP-HPLC profile of B7-33_9K(Palm) (left) and MALDI-TOF-MS (right).

7. Tag-B7-33

RP-HPLC carried out as described in the methods using the elution gradient: buffer B 30-60% in 30 minutes, t= 27.943 min.

MALDI-TOF-MS $[M+H]^+$ calculated 4196.216, observed 4194.532.

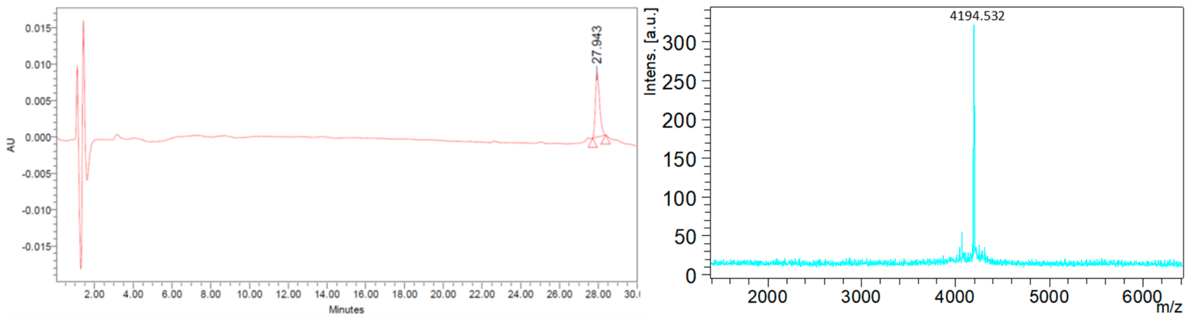

Figure S7: RP-HPLC profile of Tag-B7-33 (left) and MALDI-TOF-MS (right).

8. Tag-PEG4-B7-33

RP-HPLC carried out as described in the methods using the elution gradient: buffer B 35-65% in 30 minutes, t= 19.134 min.

MALDI-TOF-MS $[M+H]^+$ calculated 4443.766, observed 4440.682.

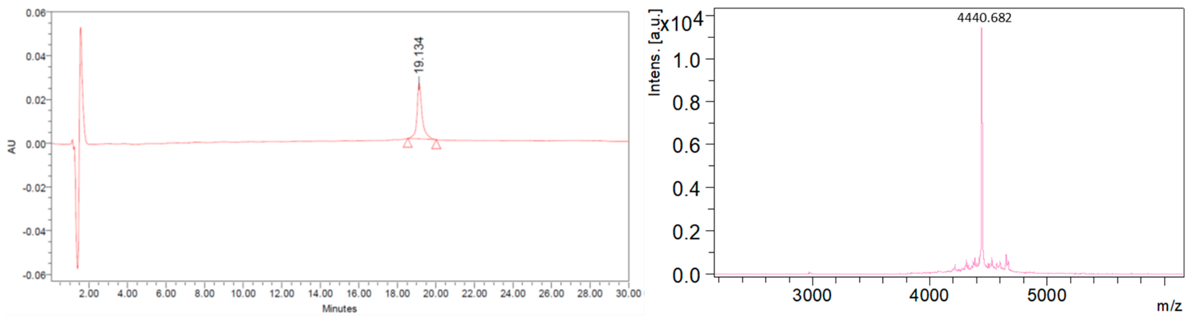

Figure S8: RP-HPLC profile of Tag-PEG4-B7-33 (left) and MALDI-TOF-MS (right).

9. Tag-PEG6-B7-33

RP-HPLC carried out as described in the methods using the elution gradient: buffer B 35-65% in 30 minutes, t= 20.079 min.

MALDI-TOF-MS $[M+H]^+$ calculated 4531.826, observed 4525.517.

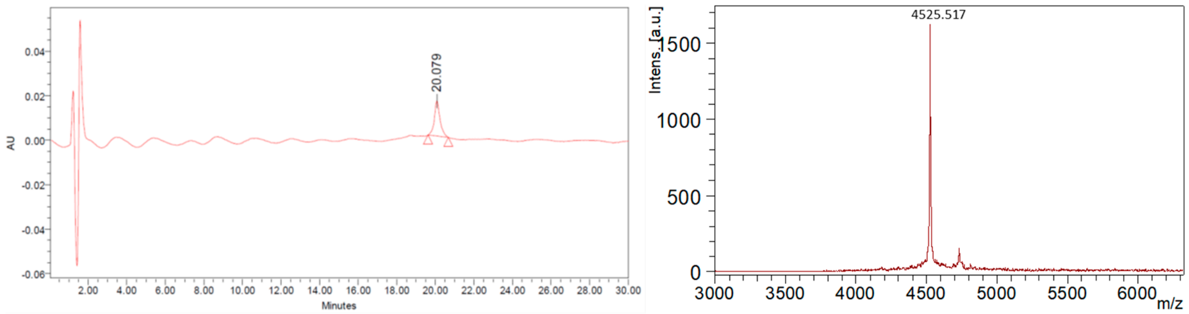

Figure S9: RP-HPLC profile of Tag-PEG6-B7-33 (left) and MALDI-TOF-MS (right).

10. K(Palm)-(PEG6)2-B7-33

RP-HPLC carried out as described in the methods using the elution gradient: buffer B 35-65% in 30 minutes, t= 17.790 min.
MALDI-TOF-MS $[M+H]^+$ calculated 4024.625, observed 4020.545.

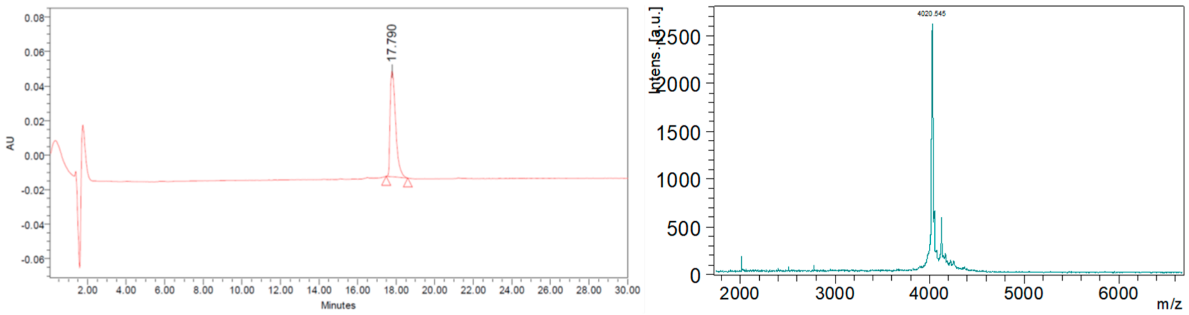

Figure S10: RP-HPLC profile of K(Palm)-(PEG6)2-B7-33 (left) and MALDI-TOF-MS (right).

11. PA-(PEG6)2-B7-33

RP-HPLC carried out as described in the methods using the elution gradient: buffer B 35-65% in 30 minutes, t= 21.220 min.
MALDI-TOF-MS $[M+H]^+$ calculated 3896.195, observed 3902.283.

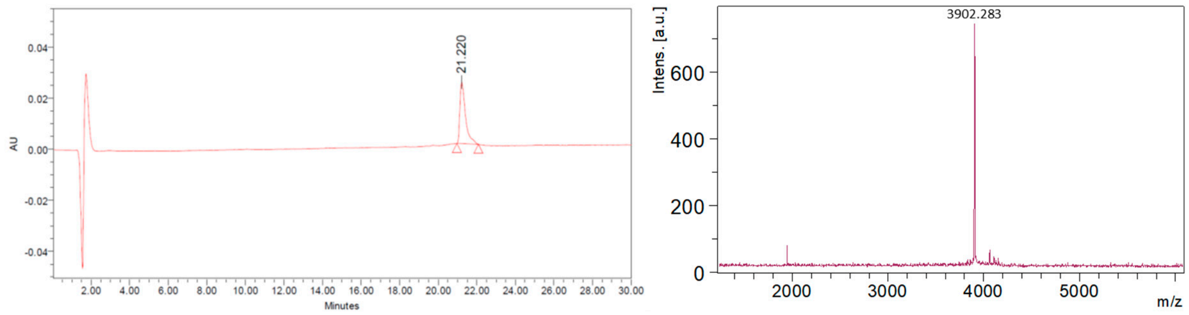

Figure S11: RP-HPLC profile of PA-(PEG6)2-B7-33 (left) and MALDI-TOF-MS (right).

12. K(Palm)-PEG12-B7-33

RP-HPLC was carried out as described in the methods using the elution gradient: buffer B 30-60% in 30 minutes, t= 21.965 min.
MALDI-TOF-MS $[M+H]^+$ calculated 3953.365, observed 3953.897.

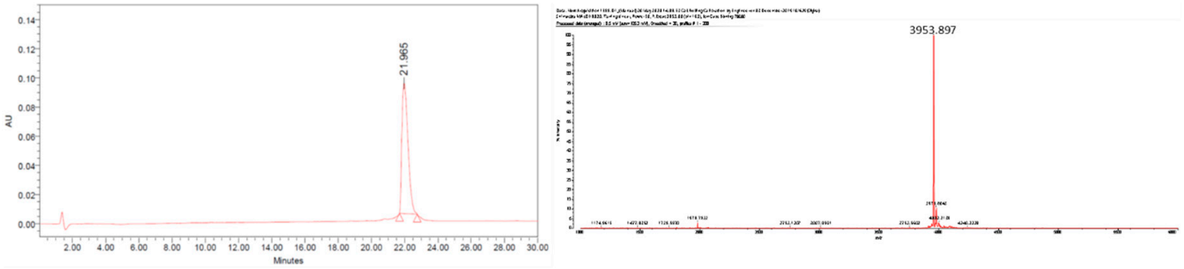

Figure S12: RP-HPLC profile of K(Palm)-PEG12-B7-33 (left) and MALDI-TOF-MS (right).

---

13. K(PalmGlu)-PEG12-B7-33

RP-HPLC carried out as described in the methods using the elution gradient: buffer B 30-60% in 30 minutes, t= 23.073 min.
MALDI-TOF-MS $[M+H]^+$ calculated 4082.506, observed 4080.542.

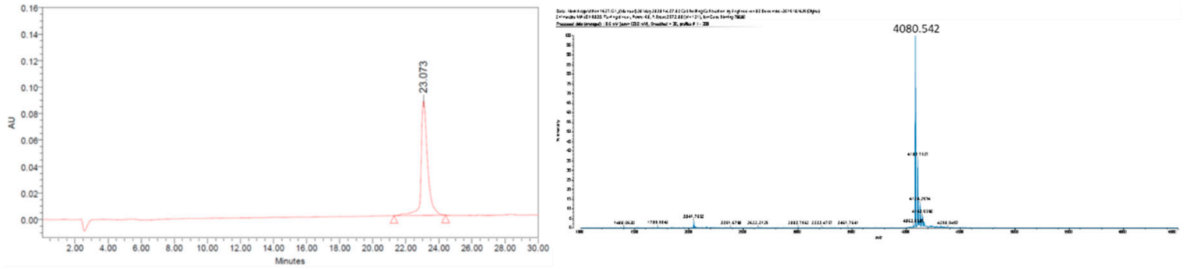

Figure S13: RP-HPLC profile of K(PalmGlu)-PEG12-B7-33 (left) and MALDI-TOF-MS (right).

---

14. AcK(PalmGlu)-PEG12-B7-33

RP-HPLC carried out as described in the methods chapter 2 using the elution gradient: buffer B 35-65% in 30 minutes, t= 19.392 min.
MALDI-TOF-MS $[M+H]^+$ calculated 4124.517, observed 4125.935.

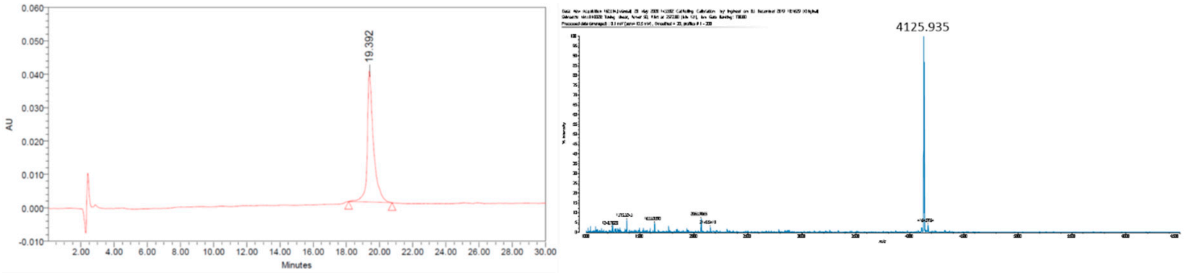

Figure S14: RP-HPLC profile of AcK(PalmGlu)-PEG12-B7-33 (left) and MALDI-TOF-MS (right).

Table S1: Summary of peptide characterization by MALDI-TOF MS and HPLC

Analogues	MALDI TOF MS		HPLC	Purity	Yield (%)
	Observed*	Calculated	t_R (min)	(%)	
B7-33	2990.834	2986.555	17.081	99.09	55
Decanoic acid-PEG6-B7-33	3481.099	3475.425	12.229	100	50
Myristic acid-PEG6-B7-33	3536.314	3532.535	17.365	99.57	45
Palmitic acid-PEG6-B7-33	3559.294	3560.585	20.33	99.92	40
K(Palm)-PEG6-B7-33	3691.177	3689.015	23.823	100	45
B7-33_9K(Palm)	3229.264	3225.231	26.347	97.34	25
Tag-B7-33	4194.532	4196.216	27.943	97.68	25
Tag-PEG4-B7-33	4440.682	4443.766	19.134	99.7	33
Tag-PEG4-B7-33	4525.517	4531.826	20.079	99.69	30
K(Palm)-(PEG6)2-B7-33	4020.545	4024.625	17.790	99.68	55
PA-(PEG6)2-B7-33	3902.283	3896.195	21.22	99.69	35
K(Palm)-PEG12-B7-33	3953.897	3953.365	21.965	99.94	42
K(PalmGlu)-PEG12-B7-33	4080.542	4082.506	23.073	99.72	45
AcK(PalmGlu)-PEG12-B7-33	4125.935	4124.517	19.392	100	45

*Observed molecular weights were determined by MALDI-TOF MS of the purified peptides
